# Psychometric test of the Team Climate Inventory-short version investigated in Dutch quality improvement teams

**DOI:** 10.1186/1472-6963-9-126

**Published:** 2009-07-24

**Authors:** Mathilde MH Strating, Anna P Nieboer

**Affiliations:** 1 Erasmus Medical Centre, Institute of Health Policy and Management, PO Box 1738, 3000 DR Rotterdam, the Netherlands

## Abstract

**Background:**

Although some studies have used the Team Climate Inventory within teams working in health care settings, none of these included quality improvement teams. The aim of our study is to investigate the psychometric properties of the 14-item version of the Team Climate Inventory in healthcare quality improvement teams participating in a Dutch quality collaborative.

**Methods:**

This study included quality improvement teams participating in the Care for Better improvement program for home care, care for the handicapped and the elderly in the Netherlands between 2006 and 2008. As part of a larger evaluation study 270 written questionnaires from team members were collected at baseline and 139 questionnaires at end measurement. Confirmatory factor analyses, reliability, Pearson correlations and paired samples t-tests were conducted to investigate construct validity, reliability, predictive validity and temporal stability.

**Results:**

Confirmatory factor analyses revealed the expected four-factor structure and good fit indices. For the four subscales – vision, participative safety, task orientation and support for innovation – acceptable Cronbach's alpha coefficients and high inter-item correlations were found. The four subscales all proved significant predictors of perceived team effectiveness, with participatory safety being the best predictor. As expected the four subscales were found to be stable over time; i.e. without significant changes between baseline and end measurement.

**Conclusion:**

The psychometric properties of the Dutch version of the TCI-14 are satisfactory. Together these results show that the TCI-14 is a useful instrument to assess to what extent aspects of team climate influence perceived team effectiveness of quality improvement teams.

## Background

Recent debates have shown great concern for the quality of health care as well as for the great variability of quality of care between care providers [1-3]. As a reaction to the gap between best practices and actual practices, which in the health care literature is referred to as the "quality chasm" [4], quality improvement collaboratives have received substantial attention as one way to close this gap. To improve a specific subject area of care temporary improvement teams from different organisations are brought together in a collaborative, allowing for learning within and between settings. Quality collaboratives are expected to enhance quality and efficiency of care by acting as a 'learning laboratory' [5] and stimulating and implementing innovations.

A team's innovativeness and thereby performance may be facilitated or hindered by the 'climate' in the team. According to one of the leading theories on innovation climate for teams, West's four-factor model [6], innovations generated by teams are influenced by vision, participative safety, task orientation and support for innovation. Teams whose members agree upon clear and realistic objectives, participate in decision making, are committed to achieve the highest possible standards of task performance, and receive support for innovative ideas, are more likely to develop new ideas and working methods.

To measure these four factors that together cover the concept of team climate Anderson and West developed the Team Climate Inventory (TCI) [7]. In several studies across different samples and countries [8-12], the psychometric properties of the instrument have shown to be acceptable. Results of these studies indicated acceptable reliability in terms of internal homogeneity, and factor analyses also confirmed the underlying four-factor structure that was hypothesized by West's four-factor theory of innovation. The original 38-item version of the TCI was shortened by Kivimäki and Elovainio [9] to a 14-item version, which also demonstrated acceptable reliability and validity. The TCI has been translated into several languages (Swedish [8]), Finnish [9], Italian [10], Norwegian [11] and German [12]. A version in Dutch was not available in the literature at the onset of this study. A first purpose of the present study was therefore to test the psychometric properties of the Dutch version of the TCI.

Although some studies have used the TCI within teams working in health care settings [9,10,13], none of these included quality improvement teams participating in a quality collaborative. Improvement teams in quality collaboratives distinguish themselves by generating and developing new ideas and behaviours and recommendations for improvement, as opposed to work teams, which implement the proposed changes. Since members of improvement teams are chosen across the organisation, division and existing work teams, members of improvement teams are not all members of the same work team. Furthermore, improvement teams are temporary, have a predefined task and goal and have the opportunity to collaborate and share knowledge and experiences with other improvement teams outside their organisation.

Applying the four-factor theory of innovation to these quality improvement teams leads to the hypothesis that vision, participative safety, task orientation and support for innovation determine the extent to which these quality improvement teams are able to think of new ways and methods to improve care processes and are more effective in improving quality of care. The purpose of this study is to empirically test the theoretical hypothesis of the four-factor structure of the TCI for improvement teams participating in a quality collaborative working on developing and implementing innovative products, working methods and services.

## Methods

### Setting and design

This study included team members of quality improvement teams participating in the quality collaborative of the Care for Better program for home care, care for the handicapped and the elderly in the Netherlands between 2006 and 2008 (for a more detailed description see [14]). These quality improvement teams were participating in the following projects: pressure ulcers, eating and drinking, prevention of sexual abuse, medication safety, fall prevention, aggression and behavioural problems and autonomy. The collaborative used the Breakthrough method as major instrument to quickly spread evidence-based practices across care organisations and enable mutual learning across sites. Although the topics of improvement were different for these projects, the set up of the projects, working with the Plan-Do-Study-Act cycle and starting off with small scale changes first, is the same.

Each improvement team consists on average of one project leader and four other team members. Two months after the start and at the end of each Breakthrough project each team member received a postal questionnaire as part of a larger overall evaluation study. For this study data from three samples were used. The first sample consisted of data from baseline questionnaires (T0) for still running projects and for which no end-measurement data are available yet. In total 125 teams received a questionnaire. Project leaders of 79 teams filled in the baseline questionnaire (T0), resulting in a response rate of 63.2% for project leaders. In total 219 other team members filled in the questionnaire. Response rate of the other team members is difficult to estimate, since no accurate data on the number of team members of teams whose project leader did not fill in the questionnaire were available. The response rate of members of teams whose project leaders did fill in the questionnaire was 62%. After excluding respondents with missing values on the TCI items a total sample of 270 respondents was left for analysis with the T0-sample.

The second sample consisted of end measurement data (T1) only, since several projects already started before the evaluation started and baseline measurement therefore was impossible. In total 83 teams received an end measurement questionnaire. Project leaders of 38 teams filled in the questionnaire, resulting in a response rate of 45.8%. This lower response rate is partly due to the fact that improvement teams participating in projects on pressure ulcers, eating and drinking, and prevention of sexual abuse were not informed beforehand about the evaluation study. In total 101 other team members filled in the questionnaire. A total sample of 139 respondents was left for analysis with the T1-sample.

The third sample consisted of respondents who filled in the baseline questionnaire, as well as the end measurement questionnaire. At this point of the evaluation study only two projects could be studied from baseline until the end of the project. Therefore, this third sample consisted of 38 respondents for whom baseline and end measurement data were available.

### Measures

The 14-item Team Climate Inventory (TCI) was assessed at baseline and end measurement (see Appendix 1). Two researchers independently had translated this instrument into Dutch. Comparison of the two translations revealed no salient differences and the two researchers agreed upon the final Dutch translation. The TCI has a 5-point response scale from 'strongly disagree' to 'strongly agree', in which higher scores indicate a better or more desirable team climate. Scores for each item in a scale are summed to determine the scale score.

In the end measurement questionnaire, team members' perceived team effectiveness was assessed by the 'perceived effectiveness' measurement instrument of Lemieux-Charles [15]. Four questions, each using a 5-point response scale, assessed the extent to which team members: (1) believed that their team's overall performance met their expectations; (2) were satisfied with their experience as a team member; (3) felt positive about their experience; and (4) would be willing to work on a similar team in the future. The reliability of the total scale was 0.76.

### Statistical analyses

The psychometric analyses comprised three parts. First, the psychometric properties of the Dutch version of the 14-item TCI were investigated by evaluating construct validity. Construct validity was analysed by conducting a confirmatory factor analysis using the LISREL program [16] to test whether or not the items loaded on the intended dimension. Each subset of measured items was allowed to load only on its corresponding latent variable derived from the four-factor theory (i.e. vision, participatory safety, task orientation and support for innovation). No correlation errors either within or across sets of items were allowed in the model, which approach is in line with previous studies of Anderson & West [7] and Kivimäki and Elovainio [9]. Furthermore, in line with the theoretical assumptions of the measurement instrument non-zero correlations between the four factors were allowed.

To test the measurement models in LISREL four indices of model fit were used. The cut-off criteria for these four indices were those proposed by Hu and Bentler [17]. First, the overall test of goodness-of-fit assesses the discrepancy between the model implied and the sample covariance matrix by means of a normal-theory weighted least squares test. A plausible model has low, preferably non-significant χ^2 ^values. However, Chi-square is overly sensitive when the sample size is large (anything over 200 [18]), leading to difficulty in obtaining desired non-significant levels [19]. Secondly, the Root Means Square Error of Approximation (RMSEA) reflects the estimation error divided by the degrees of freedom as a penalty function. Values on RMSEA below 0.06 indicate small differences between the estimated and observed model. Thirdly, we used the Standardized Root Means square Residual (SRMR), which is a scale invariant index for global fit that ranges between 0 and 1. Values on SRMR lower than 0.08 indicate a good fit. As a fourth index of model fit the Incremental Fit Index (IFI) was calculated. This index compares the independence model (i.e. observed variables are unrelated) to the estimated model. Preferably, values on IFI should be larger than 0.95.

The second part consisted of reliability analyses of the subscales. Internal consistency of the subscales emerging from the factor analysis was assessed by calculating Cronbach's alpha coefficients. After having defined the structure of the questionnaire and reliability of the subscales, Pearson's moment correlations between the factors identified by the factor analysis were computed and descriptive statistics were analysed.

The third part consisted of investigation of predictive validity and temporal stability. In order to further investigate predictive validity of the TCI, correlations of each subscale with perceived effectiveness were calculated based on the T1-sample. Furthermore, univariate regression analyses were performed. Temporal stability was analysed by conducting paired samples t-tests for each subscale based on the sample of 38 respondents for whom both baseline and end measurement data were available.

## Results

### Sample characteristics

The majority of the team members that filled in the T0 or T1 questionnaire was female, which is in accordance with the gender distribution of health care professionals. The mean age was 42 years (sd 9.9) for the T0 sample and 43 years (sd 9.2) for the T1 sample. More than two thirds of the respondents had been working for more than 3 years within the organisation. Furthermore, 232 (85.9%) team members at baseline and 94 (69.1%) team members at T1 worked more than 29 hours per week. Improvement teams mainly consisted of nurses and management staff. Table 1 lists descriptive characteristics of the two samples of team members.

**Table 1:** Sample characteristics at baseline (T0) and end measurement (T1)

		T0 n = 270		T1 n = 139	
		No.	Percentage	No.	Percentage

Sex	- female	221	83.4%	105	75.5%
	- male	44	16.6%	33	23.7%
Working past	- more than 3 years	219	81.1%	115	82.7%
Working hours	- more than 29 hours	232	85.9%	96	69.1%
Position	- medical assistants	6	2.2%	7	5.5%
	- nurses	65	24.1%	18	14.1%
	- social workers	19	7.0%	1	.8%
	- medical/social specialists	27	10.0%	16	12.5%
	- management	88	32.6%	59	46.1%
	- health policy and quality staff	31	11.5%	10	8.0%
	- para-/perimedical professionals	19	7.0%	16	12.5%
	- clients	-	-	1	.8%

No. = Number of respondents

### Factor analyses

The results of the confirmatory factor analysis with LISREL based on the T0-sample showed a good fit, as indicated by RMSEA of 0.03 – well below the boundary of 0.06 – and IFI of 0.98. The Normal Theory Weighted Least Square χ^2 ^was 263.66 and significant (p = 0.0) and the SRMR was 0.05, and thus below the cut-off point of 0.08. In table 2 the standardized solution of the four-factor structure is given. Standardized loadings of the 14 items varied between .68 and .87. Confirmatory factor analysis based on the T1-sample with 139 respondents showed similar results, which are also reported in Table 2.

**Table 2:** Standardized solution in LISREL based on T0 and T1 samples (n = 270 and n = 139 respectively)

item	vision		participative safety		task orientation		support for innovation	
	T0	T1	T0	T1	T0	T1	T0	T1
1. Agreement with objectives	0.87	0.82						
2. Team's objectives clearly understood	0.77	0.86						
3. Team's objectives achievable	0.68	0.73						
4. Worth of the objectives to the organisation	0.73	0.80						
5. 'We are together' attitude			0.72	0.61				
6. People keep each other informed			0.80	0.88				
7. People feel understood and accepted			0.80	0.80				
8. Real attempts to share information			0.85	0.82				
9. Preparedness to basic questions					0.76	0.78		
10. Critical appraisal of weaknesses					0.76	0.84		
11. Building on each other's ideas					0.83	0.92		
12. Search for new ways of looking at problems							0.75	0.83
13. Time taken to develop ideas							0.75	0.82
14. Cooperation in developing and applying ideas							0.80	0.91
								
**Model fit indices**	**T0-sample**			**T1-sample**				
RMSEA	0.03			0.04				
IFI	0.98			0.99				
χ^2^	263.66 (P = 0.0)			313.07 (P = 0.0)				
SRMR	0.05			0.06				

RMSEA = Root Mean Square Error of Approximation; IFI = Incremental Fit Index;

SRMR = Standardized Root Mean square Residual

### Reliability analyses and descriptive statistics

Reliability analyses based on the T0-sample showed alpha coefficients of the four subscales between 0.73 and 0.80, indicative of satisfactory reliability for all scales of the TCI (see Table 3). Significant positive correlations between subscales were found, ranging from 0.43 to 0.63. Furthermore, the inter-item correlations within each subscale were high. The inter-item correlation between item 2 and item 4 of the vision subscale was the lowest at 0.36. Analyses based on the T1-sample with 139 respondents showed similar results, which are reported in Table 4.

**Table 3:** Reliability, inter-item and cross-scale correlations and descriptives of subscales (T0-sample).

	Alpha	Inter-item correlations (lowest-highest)	1	2	3	Mean (sd)	Min – Max
1. Vision	0.77	0.36–0.56				16.3 (1.96)	10–20
2. Participative safety	0.80	0.43–0.57	0.56**			16.5 (2.07)	10–20
3. Task orientation	0.77	0.50–0.55	0.44**	0.62**		11.2 (1.96)	4–15
4. Support for innovation	0.73	0.47–0.48	0.43**	0.55**	0.63**	10.9 (1.83)	5–15

** p < 0.01 (1-tailed); n = 270

**Table 4:** Reliability, inter-item and cross-scale correlations and descriptives of subscales (T1-sample).

	Alpha	Inter-item correlations (lowest-highest)	1	2	3	Mean (sd)	Min – Max
1. Vision	0.82	0.47–0.60				16.3 (2.10)	9–20
2. Participative safety	0.79	0.35–0.60	0.59**			16.2 (2.26)	9–20
3. Task orientation	0.83	0.54–0.68	0.50**	0.66**		11.0 (1.99)	5–15
4. Support for innovation	0.84	0.61–0.65	0.43**	0.56**	0.68**	11.0 (2.03)	6–15

** p < 0.01 (1-tailed); n = 139

As a final step in the validation of the TCI, its predictive validity was analysed by investigating univariate regression analyses. Participatory safety emerged as the best predictor of perceived effectiveness, accounting for 29% of the variance; vision and support for innovation explained respectively 23% and 24% of the variance. Task orientation, which explained 18% of the variance, appeared to be the poorest predictor.

Temporal stability was analysed by conducting paired samples t-test for the group of 38 respondents for whom baseline and end measurement data were available. Between T0 and T1 no significant changes were found for each of the four subscales (see Table 5).

**Table 5:** Descriptive statistics TCI subscales on T0 and T1 (n = 38).

	T0		T1		Change (T0–T1)		
	Mean	SD	Mean	SD	Mean	SD	95% CI
1. Vision	16.37	2.11	16.45	1.64	-0.08	2.45	-0.88 – 0.73
2. Participative safety	16.73	2.01	16.63	1.82	0.11	2.53	-0.72 – 0.93
3. Task orientation	11.00	1.62	11.24	1.57	-0.24	2.11	-0.93 – 0.46
4. Support for innovation	11.14	1.54	11.45	1.51	-0.30	2.12	-1.00 – 0.40

95% CI = 95% confidence interval

## Discussion

In this study the psychometric properties of the TCI-14, a short measurement instrument to assess team climate, were presented. It is one of the first studies in which this instrument is studied exclusively in quality improvement teams and for the first time within a Dutch sample. Since quality improvement teams are a distinct type of team, with different structure and processes than other types of health care teams, research is needed to identify if they are effective, and under which conditions [20]. The purpose of our study was to empirically test the theoretical hypothesis of the four-factor structure of the TCI for improvement teams participating in a quality collaborative working on developing and implementing innovative products, working methods and services.

The results showed that the four-factor solution for this short version, as initially found in previous studies [9,21], is found again in a Dutch sample of team members of improvement teams in quality collaboratives. The alpha coefficients for the four subscales are acceptable, but somewhat lower than those reported by Kivimäki and Elovainio [9], which were between 0.79 and 0.86. They are comparable, however, to those found in the study of Loo and Loewen [21] at two administrations; i.e. between 0.70 and 0.80 and between 0.76 and 0.82, respectively.

As suggested by Anderson and West [7], the dimensions of the TCI may not only correlate with the number of innovations or perceived innovativeness, but also with other outcome measures such job satisfaction [22] and customer satisfaction [11]. The TCI is more and more used to assess to what extent aspects of team climate predict success or failure of strategies pursued by quality improvement teams. Support for predictive validity of the TCI subscales was found in higher scores on all scales being related with higher scores on perceived effectiveness of team members. Participatory safety was the best predictor, indicating that if team members feel they can participate in decision-making procedures, share information and feel safe to present new ideas, they will perceive a higher effectiveness of their team. Further research is needed to investigate to what extent the four dimensions of the TCI also predict more objective quality improvement outcomes such as reduction in pressure ulcers or fall incidents and client satisfaction with care.

The temporary nature of these teams may have influenced the results. Individuals participating in quality improvement teams usually have not been working together before. It may have been difficult for them, therefore, to respond to specific items. In this study, however, we deliberately sent the baseline questionnaire no earlier than two months after start of a project, so that they at least had some working experience together.

Although we had combined baseline and end-measurement data for only 38 respondents at the time of writing, our results showed that the four subscales remained stable over time. No significant changes between T0 and T1 were expected, for that matter, since the interventions within the Care for Better program were focused on improving processes of care and not on team processes and team climate. Furthermore, as explained above, the baseline questionnaire was completed not earlier than two months after start of a project, which gave improvement teams time to think about who should be in the team and come to an agreement about objectives, task division and project management. Since the TCI is more and more being used to identify areas for improvement in team functioning and to evaluate whether teams are capable of improving these areas, temporal stability of the TCI should be analyzed. The more so as this issue is still underdocumented. Loo and Loewen [21] investigated the temporal stability in management undergraduates, but not in improvement teams in health care.

The testing of theoretical associations between constructs such as team climate and team effectiveness can be analysed at the team level taking into account the hierarchical structure of the data for individuals nested within teams. As there is the potential for considerable variation within teams and since the main purpose of our study was to compare the psychometric properties of the TCI in quality improvement teams with those from previous studies on the TCI, we performed confirmatory factor analyses on the individual level. Ignoring the hierarchical structure of the data may lead to a worse fit of the model [23,24]. The factor loadings found with the two methods (individual versus team level) will be similar in value. However, in the team level model the standard errors of these estimates will be lower than in the individual level model, which in turn leads to a more adequate fit of the multilevel model. Since the team sizes are relatively small in our sample, bias of the parameter estimates and fit statistics will be less [23].

## Conclusion

To conclude, the psychometric properties of the Dutch version of the TCI-14 are satisfactory, and this short instrument is useful for assessing team climate in quality improvement teams in healthcare. This study showed that the dimensions of the TCI were predictors of perceived effectiveness of quality improvement activities. Together these results showed that the TCI is a useful instrument to assess to what extent aspects of team climate influence perceived team effectiveness.

## Abbreviations

CI: Confidence Interval; IFI: Incremental Fit Index; No.: Number; RMSEA: Root Mean Square Error of Approximation; sd: standard deviation; SRMR: Standardized Root Mean square Residual; T0: baseline; T1: end measurement; TCI: Team Climate Inventory; TCI-14: Team Climate Inventory 14 items

## Competing interests

The authors declare that they have no competing interests.

## Authors' contributions

Both authors formulated the research question and design of the study. MS carried out the study, performed the statistical analyses and drafted the manuscript. AN advised on the analyses and both authors critically revised the manuscript. Both authors read and approved the final manuscript.

## Appendix. Team climate inventory items

### Vision

1. How far are you in agreement with these objectives?

2. To what extent do you think your team's objectives are clearly understood by other members of the team?

3. To what extent do you think your team's objectives can actually be achieved?

4. How worthwhile do you think these objectives are to the organisation?

### Participative safety

5. We have a "we are in it together" attitude

6. People keep each other informed about work-related issues in the team

7. People feel understood and accepted by each other

8. There are real attempts to share information throughout the team

### Task orientation

9. Are team members prepared to question the basis of what the team is doing?

10. Does the team critically appraise potential weaknesses in what it is doing in order to achieve the best possible outcome?

11. Do members of the team build on each other's ideas in order to achieve the best possible outcome?

### Support for innovation

12. People in this team are always searching for fresh, new ways of looking at problems

13. In this team we take the time needed to develop new ideas

14. People in the team cooperate in order to help develop and apply new ideas

## Pre-publication history

The pre-publication history for this paper can be accessed here:


